# The Current Status of Minimally Invasive Conduit Harvesting for Coronary Artery Bypass Grafting

**DOI:** 10.3390/jcdd11070188

**Published:** 2024-06-23

**Authors:** Devon Anderson, Bob Kiaii, Jorge Catrip

**Affiliations:** Division of Cardiac Surgery, Department of Surgery, University of California Davis Medical Center, Sacramento, CA 95817, USA; bkiaii@ucdavis.edu (B.K.); jcatrip@ucdavis.edu (J.C.)

**Keywords:** coronary artery bypass grafting surgery, minimally invasive, conduit, cardiac surgery

## Abstract

The harvesting of conduits for coronary artery bypass surgery has evolved over the last decade to include endoscopic approaches to access the saphenous vein, radial artery, and internal mammary artery. These minimally invasive techniques reduce the morbidity associated with open procedures by decreasing pain and recovery time and increasing mobility post operatively. This review highlights the differences in morbidity, quality, and patency between the most common conduits that are harvested minimally invasively for coronary artery bypass grafting surgery.

## 1. Introduction

Coronary artery disease remains the most common heart disease in the United States and continues to impact the lives of millions of American yearly [1,2]. Coronary artery bypass grafting (CABG) surgery is the surgical revascularization procedure used to address this condition and is the most common cardiac surgical procedure performed in the world [1]. Traditionally, the harvesting of conduits for this procedure has been performed using an open technique; however, over the last two decades there has been an increased adoption of minimally invasive and endoscopic approaches to obtain the various conduits for coronary artery bypass grafts. The goal of this transition has been to reduce the morbidity of open procedures by decreasing pain and recovery time and increasing mobility post operatively, all of which has ultimately led to increased patient satisfaction [1,3,4]. This review highlights the differences in morbidity, quality, and patency in conduits that are harvested minimally invasively for coronary artery bypass grafting surgery.

## 2. Endoscopic Saphenous Vein Harvesting

The greater saphenous vein is the second most widely harvested conduit used during coronary artery bypass surgery, which can be attributed to its accessibility and the ability to harvest long segments [5]. These conduits can be anastomosed to coronary arteries with a lesser degree of native artery stenosis, which ideally would be avoided if utilizing arterial conduits. Despite these positive features, the greater saphenous vein’s durability and patency has been shown to be inferior compared to arterial conduits, which can be attributed to endothelial hyperplasia or damage to the endothelial lining during the harvesting technique or during reperfusion with higher arterial pressure [6,7].

Greater saphenous vein grafts were originally harvested through a long skin incision, which contributed to longer hospital stays due to the increased incidence of wound infections and pain and subsequently decreased patient satisfaction [5]. Endoscopic subcutaneous greater saphenous vein harvesting was first described in 1996 in response to the increased interest in minimally invasive surgery at the time [8]. The ROOBY randomized trial in 2010 performed a sub-analysis on the graft patency of endoscopic vein harvesting versus open vein harvesting in patients undergoing on- and off-pump CABG and found that saphenous veins that were endoscopically harvested had a statistically significant lower patency rate than the veins that were harvested openly; 74.5% vs. 85.2%, *p* < 0.0001 [9]. They also found a higher 1-year revascularization rate in the group of patients who had endoscopic harvesting of their saphenous vein versus open harvesting (6.7% vs. 3.4%, *p* < 0.05) [9]. The outcomes of this particular study could be secondary to the method by which the greater saphenous vein grafts were harvested endoscopically, by utilizing carbon dioxide to insufflate the subcutaneous cavity, to the use of bipolar cautery with potential thermal injury, or to the longer manipulation times with the rigid scope [9,10].

Later in 2019, the REGROUP trial, a randomized controlled trial, evaluated clinical outcomes in 1150 patients who were randomized to either endoscopic or open vein harvesting and did not show a significant difference in the rate of major adverse cardiac events amongst the two groups [10]. In addition, this trial showed a decreased incidence of leg infections in the endoscopic harvesting group (1.4%) vs. the open harvesting group (3.1%) [10]. The ISMICS systematic review and consensus paper on the endoscopic harvesting of conduits for CABG by Ferdinand et al. found that wound complications and wound infections were significantly reduced with endoscopic harvesting versus the traditional open harvesting of vein conduits after performing a pooled analysis that included over 1300 patients [1]. Based on their findings, they also recommended endoscopic saphenous vein and radial artery harvesting as the standard of care over open harvesting due to noninferiority in respect to patency rates, the quality of the conduit, and major adverse cardiac events [1]. Thus, the comparable long-term outcomes, in conjunction with decreased harvesting site complications, contributed to the adoption of the endoscopic harvesting technique for the saphenous vein grafts, despite concerns regarding increased costs [1,10,11]. However, cost analyses have shown that the cumulative costs are not statistically different between the open and endoscopic harvesting technique, as the higher equipment-related costs in the operating room associated with endoscopic harvesting are outbalanced by the costs associated with managing harvest site complications with the open harvesting technique [11,12,13].

Advancements in endoscopic harvesting have led to the “no touch” technique, which decreases the manipulation of the graft by harvesting the saphenous vein with a pedicle of surrounding perivascular tissue [14,15]. Studies have also shown that saphenous vein grafts with perivascular tissue left intact have superior levels of nitric oxide production, which may contribute to improved patency rates due to the protective features of nitric oxide [16,17]. The retrospective review by Sakurai et al. found that early outcomes of saphenous vein grafts harvested with the “no touch” technique had similar pathological characteristics to grafts harvested with the original open technique, with a preservation of the wall structure, normal architecture, and smooth muscle cells [18]. A randomized longitudinal trial by Souza et al. showed statistically significant improvement in patency rates in the group who underwent the “no touch” technique compared to the traditional method of harvesting saphenous vein grafts (90% and 76%, *p* = 0.01) [14,15]. As mentioned above, all of these features provide protection against the distention of the graft once it is placed under arterial pressure, and the endothelial nitric oxide activity decreases intimal hyperplasia and atherosclerosis [15,16,17,18]. The “no touch” technique also utilizes the ultrasonic scalpel, which has been reported to reduce thermal injury and subsequent injury to the graft [18]. A table of key trials and studies can be seen in Table 1.

Despite the earlier trials showing decreased patency and increased revascularization with endoscopic saphenous vein harvesting, the ultimate key to providing the best results with this procedure is to harvest the saphenous vein atraumatically. Decreasing endothelial damage and its potential downstream consequences is highly dependent on the skill level of the operator. The comprehensive review by Krishnamoorthy et al. highlights the important aspects and features that a standardized training program should encompass in order to harvest the best quality vein, as it has been shown that the number of conduit repairs is inversely proportional to the level of expertise of the harvester [17,19]. In addition, a structured and standardized training program with a set surgical skill curriculum provides consistent training and reproducible results across all of the harvesters [17,20].

## 3. Radial Artery Endoscopic Harvesting

The known disadvantages of endothelial and medial hyperplasia that contribute to the reduction in patency of greater saphenous vein grafts, as previously described above, have paved the way for investigations into other conduit options [21]. Total arterial myocardial revascularization is a technique utilizing all arterial grafts during coronary artery bypass surgery and includes the internal thoracic artery, radial arteries, gastroepiploic arteries, and inferior epigastric arteries. There are pros and cons to each arterial conduit that are well known and have been previously described in the literature [21]. However, this section focuses on the radial artery and the endoscopic harvesting technique.

The path for endoscopic radial artery harvesting was paved by the success noted with endoscopic greater saphenous vein harvesting over the years [3]. According to the most recent 2021 ACC/AHA/SCAI guidelines for coronary artery revascularization, the current recommendation for bypass conduits in patients undergoing CABG is for the preferential use of the radial artery over the greater saphenous vein, as the conduit to the second most important, significantly stenosed, non-left anterior-descending coronary artery to improve long-term cardiac outcomes [2]. Observational studies have shown radial artery patency rates of 92% at 1 year and 80% at 5 years when the bypassed targeted vessel has over 90% native stenosis [21].

In the systematic review and ISMICS consensus statement regarding endoscopic conduit harvesting, there is a significant reduction in wound infections with endoscopic radial artery harvesting versus open radial artery harvesting, which led to a Class I recommendation for the use of endoscopic radial artery harvesting to reduce wound-related complications [1,2,22]. Although the time to harvest the radial artery endoscopically was significantly increased compared to open harvesting, the overall operative time was not statistically different [1].

Endoscopic radial artery harvesting is also associated with increased patient satisfaction compared with the open technique with regard to cosmesis and postoperative pain, again contributing to the Class I recommendation for an endoscopic approach for radial artery harvesting [1,2,21]. In addition, the length of stay was reduced with endoscopic radial artery harvesting; however, these findings were not statistically significant [1].

A known complication associated with utilizing the radial artery as a graft during CABG is that it is prone to vasospasm, especially when exposed to competitive flow. This highlights the previously mentioned point above about the careful selection of the targeted coronary vessel with severely stenotic lesions (>90%) prior to harvesting in order to mitigate competitive flow and subsequent vasospasm [3].

Additional complications that have been noted with the use of radial artery grafts are the postoperative neurologic deficits due to injury to the superficial radial or lateral antebrachial cutaneous nerves. Sensory disturbances and neurological complications have been reported at as high as 30–67% [3,23]. These symptoms are transient and self-limiting and will usually resolve with time; however, permanent neurologic impairment was quoted to be 7.4% in one study [3,24].

Overall, endoscopically harvesting the radial artery has significant benefits when compared to open harvesting of the radial artery, as reported in the literature. The radial artery is not always available for use or the most appropriate conduit for all patients; however, it is an excellent option if the patient meets all the criteria and is amenable to endoscopic harvesting.

## 4. Endoscopic Internal Mammary Artery Harvesting

Endoscopic harvesting of the internal mammary artery has also gained popularity after advancements in minimally invasive cardiac surgery. This approach is used not only in patients with single-vessel disease, but also in patients undergoing hybrid treatment with stents to non-LAD vessels [25]. Minimally invasive CABG via anterolateral thoracotomy was first described by Dr. Kolessov in 1967 [26]. Endoscopic harvesting of the internal mammary artery with a sternal sparing mini thoracotomy approach and endoscopic camera, trocars, and instruments has been defined in the literature by Hrapkowicz et al. [25]. The benefits of this type of harvesting are the improved visualization of the artery and the ability to perform a full-length dissection of the internal mammary artery proximally, which is traditionally difficult with the conventional approach. The incomplete dissection of the proximal portion of the internal mammary artery can lead to “steal syndrome” [25].

In addition to improved visualization with the endoscopic approach, there is decreased postoperative pain. Statistically significant lower pain scores and decreased requirements for opioids postoperatively have been reported in patients undergoing endoscopic harvesting of the internal mammary artery versus conventional harvesting [27]. This can be attributed to the increased pain associated with rib retraction, which is required in the conventional method for harvesting the internal mammary artery [27].

An important aspect of totally endoscopic coronary artery bypass surgery is robot-assisted left internal mammary artery harvesting. As with all endoscopic harvesting techniques, there is a tremendous learning curve that needs to be overcome prior to achieving results comparable to the standard method of harvesting. The retrospective review by Oehlinger et al. found that the time to harvest the internal mammary artery decreased from 140 min in the first 10 cases to 34 min in the last 10 cases [28]. Other studies have shown decreased average IMA harvesting times, ranging from 57.8 ± 23.2 min in one study to 64.1 min in another, with the early postoperative angiogram showing patent grafts [29,30]. The utilization of devices such as the harmonic scalpel and increased experience demonstrated a 10% improvement in performance for each doubling of cases completed, which was seen in the first 20 cases [30,31].

Nonetheless, endoscopic harvesting of the internal mammary artery provides comparable results to open internal mammary artery harvesting and carries many benefits that outweigh the longer harvesting time.

## 5. Conclusions

Minimally invasive conduit harvesting for coronary artery bypass surgery has evolved over the last decade and continues to be modified with advancements in technology. With the more widespread adoption of the various minimally invasive techniques and increased operator expertise, the current cons associated with minimally invasive harvesting can be investigated and improved over time. It is also of paramount importance for continued institutional support to provide the necessary resources to encourage the adoption and evolution of minimally invasive approaches.

## Figures and Tables

**Table 1:** Results of key trials and studies for endoscopic saphenous vein harvesting.

Author	Year	Type of Study	Results
Zenati et al. [9] (ROOBY Trial)	2010	Randomized controlled trial	Statistically significant lower patency rate in endoscopically harvested veins than veins that were harvested open; 74.5% vs. 85.2%, *p* < 0.0001 Higher 1-year revascularization rate in the group of patients who had endoscopic harvesting of their saphenous vein versus open harvesting (6.7% vs. 3.4%, *p* < 0.05)
Zenati et al. [10] (REGROUP Trial)	2019	Randomized controlled trial	No significant difference in the rate of major adverse cardiac events Leg infections occurred in 3.1% of patients in the open harvesting group and 1.4% of patients in the endoscopic harvesting group (relative risk, 2.26; 95% CI, 0.99 to 5.15)
Ferdinand et al. [1]	2017	Systematic review and meta-analysis	Odds of a wound infection were significantly reduced with endoscopic harvesting (OR = 0.28, 95% CI = 0.13 to 0.63, *p* = 0.002)
Souza et al. [14]	2006	Randomized longitudinal trial	Angiographic assessment at 18 months postoperatively showed 89% conventional versus 95% no-touch grafts were patent. Repeated angiography at 8.5 years showed a patency rate for the conventional group of 76% and 90% for the no-touch group (*p* = 0.01)
Sakurai et al. [18]	2022	Retrospective review	Similar pathological characteristics as grafts harvested with the original and no-touch technique

## Data Availability

No new data were created.

## References

[1] Ferdinand F.D., MacDonald J.K., Balkhy H.H., Bisleri G., Hwang H.Y., Northrup P., Trimlett R.H.J., Wei L., Kiaii B.B. (2017). Endoscopic conduit harvest in coronary artery bypass grafting surgery an ISMICS Systematic Review and Consensus Conference Statements. Innov. Technol. Tech. Cardiothorac. Vasc. Surg..

[2] Lawton J.S., Tamis-Holland J.E., Bangalore S., Bates E.R., Beckie T.M., Zwischenberger B.A. (2022). 2021 ACC/AHA/SCAI Guideline for Coronary Artery Revascularization: A Report of the American College of Cardiology/American Heart Association Joint Committee on Clinical Practice Guidelines. Circulation.

[3] Aziz O., Athanasiou T., Darzi A. (2006). Minimally invasive Conduit harvesting: A systematic review. Eur. J. Cardio-Thorac. Surg..

[4] Akowuah E., Burns D., Zacharias J., Kirmani B.H. (2021). Endoscopic vein harvesting. J. Thorac. Dis..

[5] Mahmood D., Rosati F., Petsikas D., Payne D., Torkan L., Bisleri G. (2019). Endoscopic saphenous vein harvesting with a non-sealed approach. Multimed. Man. Cardio-Thorac. Surg..

[6] Campeau L., Lespérance J., Hermann J., Corbara F., Grondin C.M., Bourassa M.G. (1979). Loss of the improvement of angina between 1 and 7 years after aortocoronary bypass surgery: Correlations with changes in vein grafts and in coronary arteries. Circulation.

[7] Raza S., Chang C., Deo S.V., Sabik J.F. (2018). Current role of saphenous vein graft in coronary artery bypass grafting. Indian J. Thorac. Cardiovasc. Surg..

[8] Lumsden A. (1996). Subcutaneous, video-assisted saphenous vein harvest: Report of the first 30 cases. Cardiovasc. Surg..

[9] Zenati M.A., Shroyer A.L., Collins J.F., Hattler B., Ota T., Almassi G.H., Amidi M., Novitzky D., Grover F.L., Sonel A.F. (2011). Impact of endoscopic versus open saphenous vein harvest technique on late coronary artery bypass grafting patient outcomes in the ROOBY (Randomized on/off Bypass) Trial. J. Thorac. Cardiovasc. Surg..

[10] Zenati M.A., Bhatt D.L., Bakaeen F.G., Stock E.M., Biswas K., Gaziano J.M., Kelly R.F., Tseng E.E., Bitondo J., Quin J.A. (2019). Randomized Trial of Endoscopic or Open Vein-Graft Harvesting for Coronary-Artery Bypass. N. Engl. J. Med..

[11] Rosati F., Bin Pervez M., Palacios C.M., Tomasi C., Mastroiacovo G., Pirola S., Bonomi A., Polvani G., Bisleri G. (2022). Cost Analysis of Endoscopic Conduit Harvesting Technique Using a Non-Sealed System for Coronary Artery Bypass Surgery. Innov. Technol. Tech. Cardiothorac. Vasc. Surg..

[12] Eckey H., Heseler S., Hiligsmann M. (2023). Economic Evaluation of Endoscopic vs. Open Vein Harvesting. Ann. Thorac. Surg..

[13] Rao C., Aziz O., Deeba S., Chow A., Jones C., Ni Z., Papastavrou L., Rahman S., Darzi A., Athanasiou T. (2008). Is minimally invasive harvesting of the great saphenous vein for coronary artery bypass surgery a cost-effective technique?. J. Thorac. Cardiovasc. Surg..

[14] Souza D.S., Johansson B., Bojö L., Karlsson R., Geijer H., Filbey D., Bodin L., Arbeus M., Dashwood M.R. (2006). Harvesting the saphenous vein with surrounding tissue for CABG provides long-term graft patency comparable to the left internal thoracic artery: Results of a randomized longitudinal trial. J. Thorac. Cardiovasc. Surg..

[15] Sepehripour A.H., Jarral O.A., Shipolini A.R., McCormack D.J. (2011). Does a “no-touch” technique result in better vein patency?. Interact. Cardio Vasc. Thorac. Surg..

[16] Saito T., Kurazumi H., Suzuki R., Matsunaga K., Tsubone S., Lv B., Kobayashi S., Nagase T., Mizoguchi T., Samura M. (2022). Perivascular Adipose Tissue Is a Major Source of Nitric Oxide in Saphenous Vein Grafts Harvested via the No-Touch Technique. JAHA.

[17] Krishnamoorthy B., Critchley W.R., Venkateswaran R.V., Barnard J., Caress A., Fildes J.E., Yonan N. (2016). A comprehensive review on learning curve associated problems in endoscopic vein harvesting and the requirement for a standardised training programme. J. Cardiothorac. Surg..

[18] Sakurai H., Someya T., Yamamoto S., Kasahara I., Kuroki H., Shirai T. (2022). Short-Term Evaluation of a Novel No-Touch Technique for Harvesting Saphenous Veins With Long-Shafted Ultrasonic Scalpel. Innov. Technol. Tech. Cardiothorac. Vasc. Surg..

[19] Pagni S., A Ulfe E., Montgomery W.D., VanHimbergen D.J., Fisher D.J., A Gray L., A Spence P. (1998). Clinical experience with the video-assisted saphenectomy procedure for coronary bypass operations. Ann. Thorac. Surg..

[20] Xue A., Chen S., Ranade A., Smith K., Kasten J., Catrip J., Kiaii B. (2022). How to implement a clinical robotic mitral valve surgery program. Ann. Cardiothorac. Surg..

[21] Maestri F., Formica F., Gallingani A., Gripshi F., Nicolini F. (2022). Radial Artery Versus Saphenous Vein as Third Conduit in Coronary Artery Bypass Graft Surgery for Multivessel Coronary Artery Disease: A Ten-Year Literature Review. Acta Biomed..

[22] Patel A.N., Henry A.C., Hunnicutt C., Cockerham C.A., Willey B., Urschel H.C. (2004). Endoscopic radial artery harvesting is better than the open technique. Ann. Thorac. Surg..

[23] Saeed I., Anyanwu A.C., Yacoub M.H., Amrani M. (2001). Subjective patient outcomes following coronary artery bypass using the radial artery: Results of a crosssectional survey of harvest site complications and quality of life. Eur. J. Cardiothorac. Surg..

[24] Casselman F.P., La Meir M., Cammu G., Wellens F., De Geest R., Degrieck I., Van Praet F., Vermeulen Y., Vanermen H. (2004). Initial experience with an endoscopic radial artery harvesting technique. J. Thorac. Cardiovasc. Surg..

[25] Hrapkowicz T., Bisleri G. (2013). Endoscopic harvesting of the left internal mammary artery. Ann. Cardiothorac. Surg..

[26] Kolessov V.I. (1967). Mammary artery-coronary artery anastomosis as method of treatment for angina pectoris. J. Thorac. Cardiovasc. Surg..

[27] Bucerius J., Metz S., Walther T., Falk V., Doll N., Noack F., Holzhey D., Diegeler A., Mohr F.W. (2002). Endoscopic internal thoracic artery dissection leads to significant reduction of pain after minimally invasive direct coronary artery bypass graft surgery. Ann. Thorac. Surg..

[28] Oehlinger A., Bonaros N., Schachner T., Ruetzler E., Friedrich G., Laufer G., Bonatti J. (2007). Robotic Endoscopic Left Internal Mammary Artery Harvesting: What Have We Learned After 100 Cases?. Ann. Thorac. Surg..

[29] Kodera K., Boyd W.D., Kiaii B., Novik R.J., Rayman R., Ganapathy S., Dobkowski W.B., McKenzie N.F., Menkis A.H., Otsuka T. (2001). Clinical experience in thoracoscopic left internal mammary artery harvesting with voice activated robotic assistance. Kyobu Geka.

[30] Boyd W.D., Kiaii B., Novick R.J., Rayman R., Ganapathy S., Dobkowski W.B., Menkis A.H. (2001). RAVECAB: Improving outcome in off-pump minimal access surgery with robotic assistance and video enhancement. Can. J. Surg..

[31] Hemli J.M., Henn L.W., Panetta C.R., Suh J.S., Shukri S.R., Jennings J.M., Fontana G.P., Patel N.C. (2013). Defining the Learning Curve for Robotic-Assisted Endoscopic Harvesting of the Left Internal Mammary Artery. Innov. Technol. Tech. Cardiothorac. Vasc. Surg..

